# PRMT1 alleviates isoprenaline-induced myocardial hypertrophy by methylating SRSF1: PRMT1 and myocardial hypertrophy

**DOI:** 10.3724/abbs.2024175

**Published:** 2024-12-10

**Authors:** Zi Yan, Wenhui Zhao, Naixin Zhao, Yufeng Liu, Bowen Yang, Li Wang, Jingyi Liu, Deping Wang, Jin Wang, Xiangying Jiao, Jimin Cao, Jianguo Li

**Affiliations:** 1 Department of Physiology Shanxi Medical University Taiyuan 030001 China; 2 MOE Key Laboratory of Cellular Physiology Shanxi Medical University Taiyuan 030001 China; 3 Department of Pathology Shanxi Medical University Taiyuan 030001 China; 4 Department of Cardiology Shanxi Cardiovascular Hospital Taiyuan 030001 China; 5 Guangdong Province Key Laboratory of Psychiatric Disorders Guangzhou 510515 China

**Keywords:** myocardial hypertrophy, protein arginine methyltransferase, serine/arginine-rich splicing factor 1, methylation, phosphorylation

## Abstract

Myocardial hypertrophy (MH) is an important factor contributing to severe cardiovascular disease. Previous studies have demonstrated that specific deletion of the protein arginine methyltransferase 1 (PRMT1) leads to MH, but the exact mechanism remains unclear. Serine/arginine-rich splicing factor 1 (SRSF1) affects the development and progression of cardiovascular disease by selectively splicing downstream signaling proteins. The present study is designed to determine whether PRMT1 is involved in MH by regulating SRSF1 and, if so, to explore the underlying mechanisms. Adult male mice and H9C2 cardiomyocytes are treated with isoprenaline (ISO) to establish MH models. The expression levels of PRMT1 are significantly decreased in the ISO-induced MH models, and inhibiting PRMT1 worsens MH, whereas overexpression of PRMT1 ameliorates MH. SRSF1 serves as the downstream target of PRMT1, and its expression is markedly elevated in MH. Moreover, SRSF1 increases the mRNA expressions of CaMKIIδ A and CaMKIIδ B, decreases the mRNA expression of CaMKIIδ C by altering the selective splicing of CaMKIIδ, and further participates in MH. In addition, there is an interaction between PRMT1 and SRSF1, whereby PRMT1 reduces the phosphorylation level of SRSF1 via methylation, thus further altering its functional activity and eventually improving MH. Our present study demonstrates that PRMT1 relieves MH by methylating SRSF1, which is expected to provide a new theoretical basis for the pathogenic mechanism of MH and potential drug targets for reducing MH and associated cardiovascular disease.

## Introduction

Cardiovascular disease is the number one killer of human health [1]. According to the latest epidemiologic data, the incidence and mortality of cardiovascular disease are experiencing a constantly rising phase, and two of every five deaths are from cardiovascular disease [2]. Myocardial hypertrophy (MH) is the pathologic basis of multiple major cardiovascular diseases, such as hypertension, coronary heart disease (CHD), and heart failure (HF) [3]. The currently available preventive and therapeutic means for MH are inadequate to solve all clinical problems, suggesting that some unknown pathogenic factors exist in MH.

In recent years, increasing evidence has demonstrated that epigenetic mechanisms play important roles in the pathophysiology of MH [4]. Protein arginine methylation is a common epigenetic modification that may alter gene expression by transferring methyl groups to the guanidine nitrogen atom. Protein arginine methyltransferase 1 (PRMT1) is one of the main protein arginine methyltransferases (PRMTs) [5]. Previous studies have confirmed that cardiac-specific PRMT1 deletion leads to MH [6], suggesting that PRMT1 is closely associated with the development and progression of MH, but the exact mechanism has not been identified. Research has verified that serine/arginine-rich splicing factor 1 (SRSF1), a type of selective splicing factor, contributes to the diversity and complexity of proteomics by affecting gene expression, whereas abnormal splicing may produce severe pathological consequences [7]. Although substantial evidence indicates that aberrant protein selective splicing is related to MH, in which SRSF1 is involved in cardiovascular disease by selectively splicing downstream signaling proteins [8], whether PRMT1 affects MH by regulating SRSF1 remains largely unknown.

Therefore, the aims of the present study were: 1) to observe the expression of myocardial PRMT1 in isoprenaline (ISO)-induced MH models; 2) to explore whether PRMT1 participates in the development and progression of MH by regulating SRSF1; and, if so, 3) to delineate the potential mechanisms responsible for MH.

## Materials and Methods

### Animals and ethical statement

Specific pathogen-free (SPF) healthy C57 male mice aged 6 weeks weighing 25–30 g were purchased from Beijing Changyang Xishan Breeding Farm (Beijing, China). In accordance with methods described in the literature [9], an MH mouse model was established by injecting 5 mg/kg ISO (HY-B0468; MedChemExpress, Monmouth Junction, USA) via the tail vein for two weeks. The protocols were performed in adherence with the National Institutes of Health Guidelines on the Use of Laboratory Animals and were approved by the Bioethics Committee of Shanxi Medical University (Taiyuan, China).

### Bioinformatics analysis

*PRMT1* and *MH* genes were acquired from the Gene Expression Omnibus (GEO) datasets (GSE230585 and GSE153143, respectively) and analyzed by Draw Venn Diagram ( https://bioinformatics.psb.ugent.be/webtools/Venn/) to obtain intersecting genes, followed by protein interaction analysis through the STRING database ( https://string-db.org/) and graphical analysis through Cytoscape ( http://www.cytoscape.org/). Finally, genes that were highly correlated with PRMT1 and MH were obtained.

### Echocardiography

After successful anaesthesia of the mice with 1%–2% (v/v) isoflurane, echocardiography was performed using the Vevo LAZR-X photoacoustic imaging system (VisualSonics, Toronto, Canada). Left ventricular (LV) fractional shortening (FS) and ejection fraction (EF) were automatically calculated as follows: FS%=[(LVEDD–LVESD)/LVEDD]×100% and EF%=[(LVEDV–LVESV)/LVEDV]×100%. LVEDD is the LV end-diastolic diameter (LVEDD), and LVESD is the LV end-systolic diameter (LVESD). LVEDV is the LV end-diastolic volume, and LVESV is the LV end-systolic volume.

### Measurement of body weight (BW) and heart weight (HW)

The mice were weighed and then sacrificed. Hearts were rapidly removed, trimmed to remove major blood vessels, sectioned, blotted, and then weighed.

### Histology and immunohistochemistry (IHC)

The mouse heart tissues were fixed in 4% polyformaldehyde (POM), paraffin embedded, sliced into sections, dewaxed, and rehydrated. Morphological changes and myocardial fibrosis were assessed via hematoxylin-eosin (HE) staining (DH0006; Leagene, Beijing, China) and Sirius Red staining (PH1098; Phygene, Fuzhou, China). The expressions and localization of the proteins were assayed by IHC. After antigen retrieval, the tissue sections were separately immunostained with anti-SRSF1 antibody (66671-1-Ig; Proteintech, Chicago, USA) and anti-PRMT1 antibody (ab190892; Abcam, Cambridge, UK) at 37°C for 2 h, followed by incubation with horseradish peroxidase (HRP)-labelled secondary antibody (SV0002; Boster, Wuhan, China) at 37°C for 30 min. Finally, color development was performed with DAB chromogenic reagent (AR1027; Boster).

### Cell culture, transfection, and infection

H9C2 cardiomyocytes (Suzhou Haixing Biotechnology, Suzhou, China) were cultured in Dulbecco’s modified Eagle’s medium (DMEM; Boster) supplemented with 10% fetal bovine serum (SA211; CellMax, Beijing, China). Cells were cultured in complete medium containing 10 μM SPHINX31 (HY-117661; MedChemexpress) for 24 h to inhibit SRSF1 activity. For transfections, H9C2 cells were seeded in 6-well plates at a density of 2×10 ^5^ cells/well. When the cell confluence reached 50%, transfection was performed with fresh medium containing lentivirus (Shanghai Hanheng Biotechnology, Shanghai, China) and polybrene for 24 h. The luciferase activity of the cells was assayed according to the manufacturer’s instructions.

### Immunofluorescence (IF) staining

H9C2 cardiomyocytes were washed with PBS (MA0015; Meilunbio Biological Technology, Dalian, China), fixed with 4% paraformaldehyde for 20 min, cultured in 3% H _2_O _2_ for 15 min, blocked with bovine serum albumin (BSA) buffer solution for 1 h, and then incubated with the primary antibodies overnight as follows: anti-PRMT1 antibody (ab190892; Abcam) and anti-SRSF1 antibody (SC-33652; Santa Cruz Biotechnology, Santa Cruz, USA). After that, the cells were incubated with a fluorescence-labelled secondary antibody at room temperature for 1 h, after which DAPI (AR1176; Boster) was used to stain the nuclei. Finally, the images of the cells were digitized via a fluorescence microscope (Nikon, Tokyo, Japan). Images were taken from nine different fields of a single well.

### Western blot analysis

The mouse myocardial tissues or H9C2 cardiomyocytes were lyzed with RIPA lysis buffer (AR0102-100; Boster) on ice to obtain the supernatant. The protein concentration was determined with a BCA Protein Assay kit (Boster). The protein samples were subjected to SDS-PAGE, and transferred to a PVDF membrane. The membrane was blocked with 5% skim milk for 2 h and then cultured with primary antibodies overnight, followed by incubation with an HRP-conjugated anti-rabbit IgG secondary antibody (BA1054; Boster) for 2 h. Finally, the membrane was developed and visualized with an imaging system. The primary antibodies used were as follows: anti-SRSF1 antibody (SC-33652; Santa Cruz), anti-ANP antibody (SC-515701-S; Santa Cruz), anti-PRMT1 antibody (ab190892; Abcam), anti-HSP90a antibody (A5006; ABclonal, Wuhan, China), and anti-ADMA antibody (A18262; ABclonal). In addition, the phosphorylation of SRSF1 was detected by the use of phosbind acrylamide (F4002; APExBIO, Houston, USA).

### Immunoprecipitation (IP) assay

The protein levels of PRMT1, SRSF1, and pan-ADMA were detected using a Pierce® Crosslink Immunoprecipitation kit (26147; Thermo Fisher Scientific, Waltham, USA). The mouse myocardial tissues or H9C2 cardiomyocytes were lyzed in IP lysis/wash buffer on ice to obtain the supernatant. Protein A/G agarose beads and an anti-SRSF1 antibody were added to Pierce Spin Columns for mixing. Then, the cell lysate was added to Pierce Spin Columns and cultured at 4°C overnight. On the following day, the protein samples were collected and subject to western blot analysis.

### Quantitative PCR analysis

According to the manufacturer’s instructions, total RNA was extracted from heart tissues or H9C2 cardiomyocytes using Trizol reagent (MF034; Mei5 Biotechnology, Beijing, China) on ice and reverse-transcribed into cDNA using the M5 Sprint qPCR RT kit (MF949; Mei5 Biotechnology). Quantitative PCR was performed using 2× M5 Hiper SYBR Premix EsTaq (with Tli RnaseH) (MF787; Mei5 Biotechnology). A comparative CT method was used to analyze the relative changes in gene expression. The results are expressed relative to the expression of *Gapdh* (internal control). The sequences of primers used are listed in Table 1.

**Table:** **Table 1** Sequences of the primers used for RT-qPCR

Primer	Sequence (5′→3′ )
*Prmt1* forward	GCACCCTCACATACCGCAACTC
*Prmt1* reverse	GCTGATGATGATGTCCACCTTCTCC
*Srsf1* forward	GCCCTTCGCCTTCGTTGAGTTC
*Srsf1* reverse	CCAGTGCCATCTCGGTAAACATCAG
*Anp* forward	CAGAATCGACTGCCTTTTCC
*Anp* reverse	GGGGGTAGGATTGACAGGAT
*Bnp* forward	ACCCAGGCAGAGTCAGAAAC
*Bnp* reverse	ACAAGATAGACCGGATCGGA
*CamkII* forward	CGAGAAATTTTTCAGCAGCC
*CamkIIδ A* reverse	ACAGTAGTTTGGGGCTCCAG
*CamkIIδ B* reverse	GCTCTCAGTTGACTCCATCATC
*CamkIIδ C* reverse	CTCAGTTGACTCCTTTACCCC
*Gapdh* forward	CAGTGCCAGCCTCGTCCCGATGA
*Gapdh* reverse	CTGCAAATGGCAGCCCTGGTGAC

### Fluo-4AM and immunofluorescence staining

Cardiomyocytes grown on 24-well plates were washed three times with PBS for 15 min to remove trypsin from the culture medium, incubated with Fluo-4AM (F8500; Solarbio, Beijing, China) at 37°C for 20 min and observed under a fluorescence microscope.

### TUNEL assay

The degree of cardiomyocyte apoptosis was detected with a TUNEL apoptosis assay kit (Boster) according to the manufacturer’s instructions. Briefly, H9C2 cardiomyocytes were fixed with 4% paraformaldehyde for 30 min and then digested with proteinase K at 37°C for 1 min. These samples were subsequently incubated with a TUNEL reaction mixture containing terminal deoxyribonucleotide transferase (TdT)-enzyme and biotin-dUTP at 37°C for 2 h, followed by incubation with the blocking solution at room temperature for 30 min and SABC dilution at 37°C for 30 min. The nuclei were stained with DAPI in the dark for 5 min. Finally, the TUNEL-stained cells were observed via fluorescence microscopy. The ratio of the number of TUNEL-positive nuclei to the total number of nuclei was used to indicate apoptosis. Four randomly selected fields were chosen and analyzed blindly.

### Cell surface area analysis

H9C2 cardiomyocytes were rinsed and fixed with 4% paraformaldehyde for 10 min. Next, the cells were washed and permeabilized in PBS containing 0.5% Triton X-100 (85111; Thermo Fisher Scientific) for 5 min, followed by incubation with TRITC-phalloidin for 30 min at room temperature. The nuclei were subsequently stained for 5 min with DAPI. After four rinses with PBS, the cells were sealed and photographed with a fluorescence microscope.

### Statistical analysis

Statistical analyses were performed via GraphPad Prism 8.3.0. Data are expressed as the mean ± SE. Student’s *t* test was performed to compare the differences between the two groups. When multiple groups were compared, the data were analyzed by one-way ANOVA and the least significant difference (LSD) test. All the data were analyzed in a blinded manner. All the data are representative of at least three technical repeats. *P*  < 0.05 was considered statistically significant.

## Results

### PRMT1 expression is decreased in MH, and inhibiting PRMT1 aggravates MH, whereas overexpressing PRMT1 improves MH

**Figure 1:**
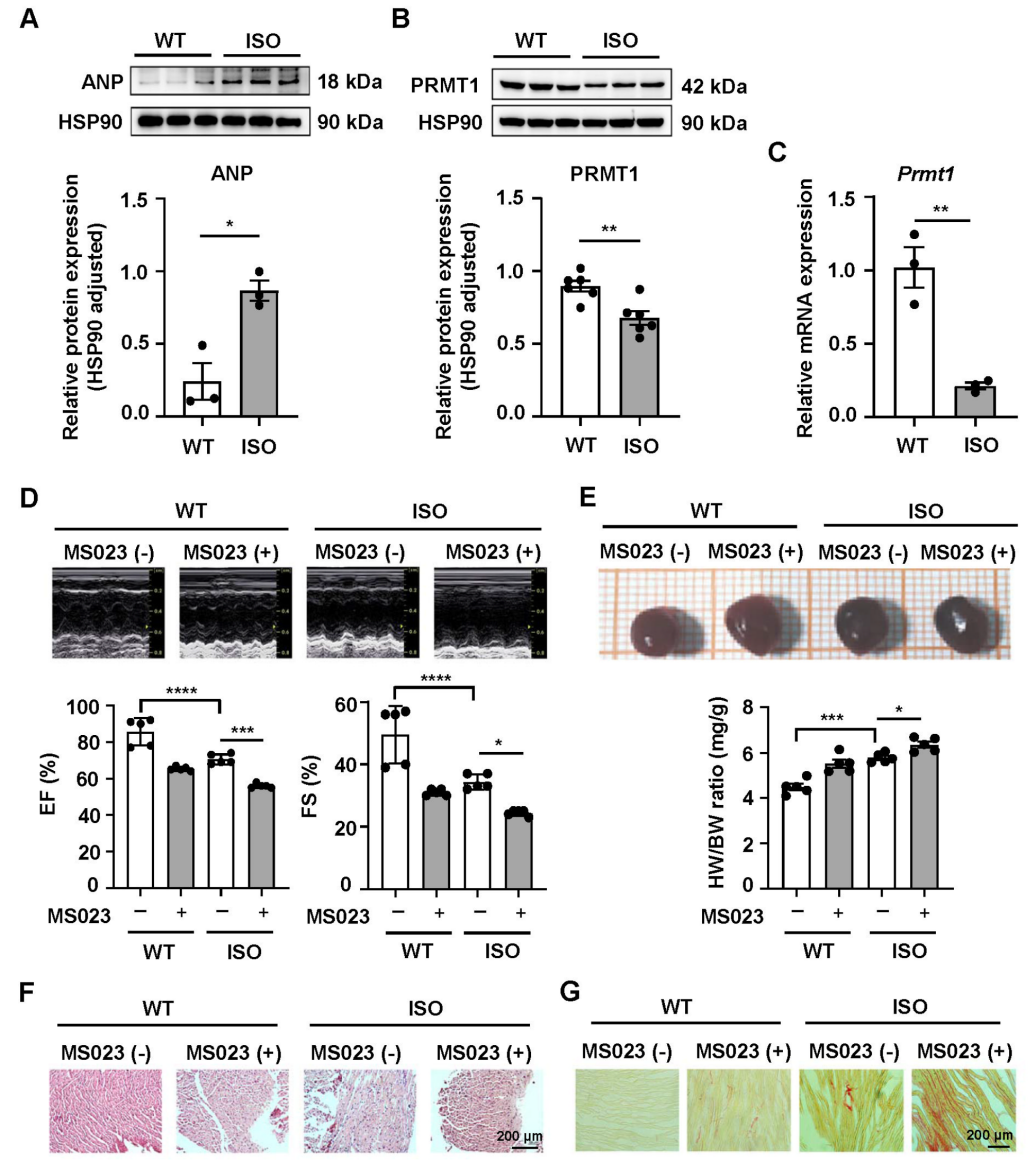
Figure 1 The expression of PRMT1 decreases in the context of myocardial hypertrophy, and PRMT1 inhibition aggravates ISO-induced myocardial hypertrophy in mice (A,B) Western blot analysis of ANP and PRMT1 protein levels in the myocardial tissues of wild-type (WT) and ISO-treated mice. (C) Quantitative PCR analysis of the mRNA expression of myocardial Prmt1 in WT and ISO-treated mice. (D) Short-axis transthoracic M mode echocardiographic tracings from WT and ISO-treated mice. (E) Photograph of hearts from 8-week-old mice. The relative heart weight (HW) of the ISO-induced mice is normalized to the body weight (BW). (F) H&E staining of mouse myocardial tissues. Scale bar: 200 μm. (G) Sirius red staining of myocardial tissues from the mice. Scale bar: 200 μm. n = 3‒6 mice/group. Data are presented as the mean ± SE. *P < 0.05, **P < 0.01, ***P < 0.001, ****P < 0.0001. EF, ejection fraction; FS, fraction shortening.

To observe the changes in the expression of PRMT1 in myocardial tissues with MH, an MH model was established by injection of ISO via the mouse tail vein. The results of the western blot analysis revealed that the protein expression of atrial natriuretic peptide (ANP) in the myocardial tissues of the ISO group was significantly increased ( Figure 1A), and the protein expression of PRMT1 was obviously decreased ( Figure 1B). Quantitative PCR analysis revealed that the mRNA expression of *Prmt1* in the myocardial tissue of the ISO group was markedly decreased ( Figure 1C). These findings indicated that both the protein and the mRNA expressions of PRMT1 were decreased in the myocardial tissues of the ISO group.

To explore the role of PRMT1 changes in MH, echocardiography was performed in ISO-induced MH mice aged 6 weeks. In ISO-induced MH mice, the left ventricular diameter (LVD) was increased significantly, and administration of the type I PRMT inhibitor MS023 exacerbated the ISO-induced increase in LVD. PRMT1 inhibition also intensified the ISO-induced decrease in the left ventricular ejection fraction (EF) and fractional shortening (FS) ( Figure 1D). Mass analysis revealed that the relative heart weight (HW) and body weight (BW) ratio (HW/BW) was increased in the ISO group, and inhibiting PRMT1 further increased this ratio ( Figure 1E). Histological analysis revealed that the morphology of left ventricular myocardial cells was significantly increased in the ISO group and further increased in the ISO + PRMT1 inhibition group ( Figure 1F). We then analyzed myocardial fibrosis in heart sections via Sirius Red staining, and the qualitative results revealed that the accumulation of collagen in the ISO + PRMT1 inhibition group was greater than that in the ISO group ( Figure 1G).

**Figure 2:**
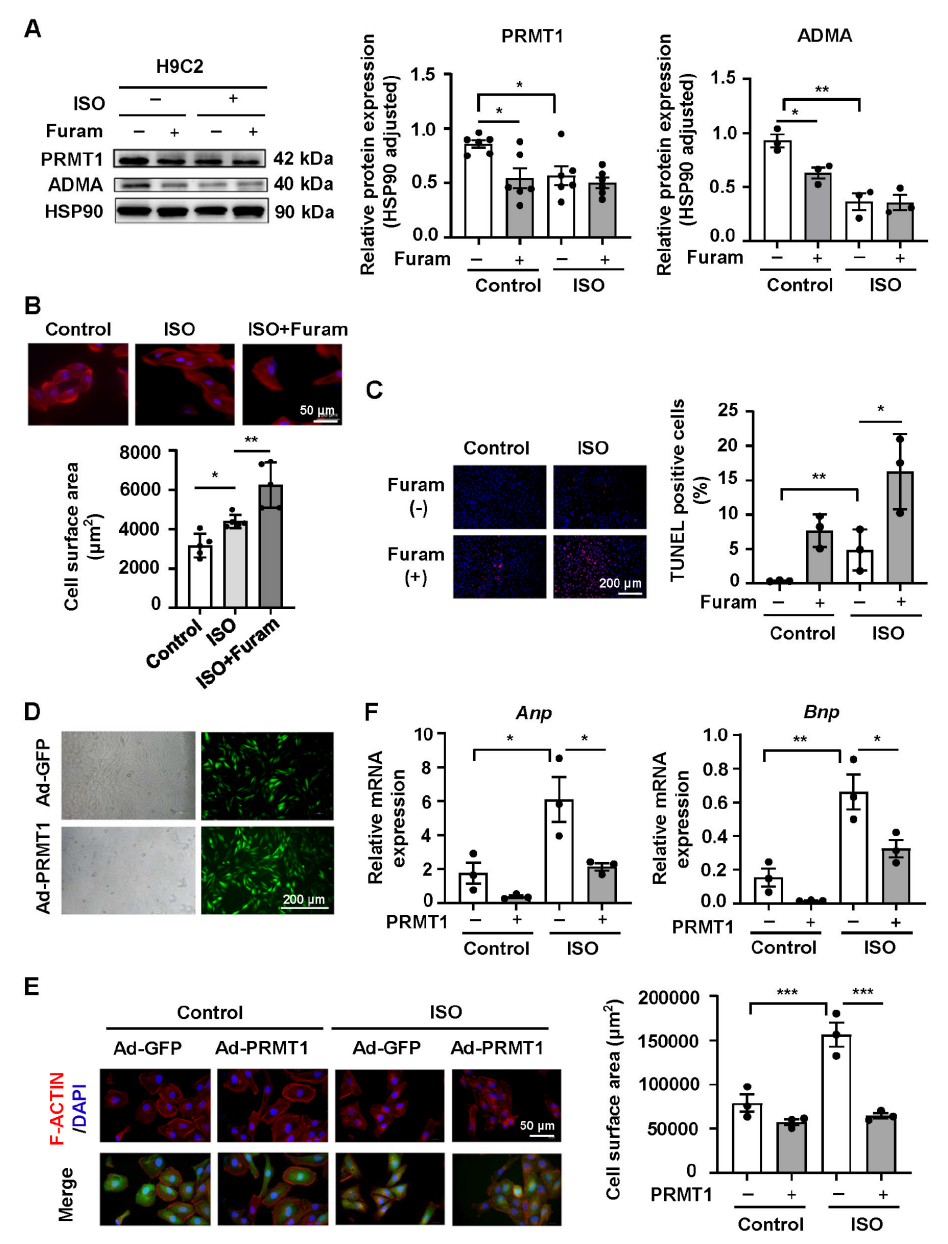
Figure 2 Specific inhibition of PRMT1 intensifies ISO-induced myocardial hypertrophy, whereas overexpression of PRMT1 attenuates myocardial hypertrophy in H9C2 cardiomyocytes (A) Western blot analysis of the expression of PRMT1 and its methylation product ADMA in cardiomyocytes. (B) The cell surface area of cardiomyocytes treated with the PRMT1 inhibitor furamidine is shown by F-actinin staining (red). Scale bar: 50 μm. (C) Representative microscopy images of TUNEL-positive nuclei (red) in cardiomyocytes treated with the PRMT1 inhibitor furamidine and quantification of apoptotic cells. Scale bar: 200 μm. (D) Representative immunofluorescence images of cardiomyocytes infected with Ad-GFP or Ad-PRMT1. (E) The cell surface area of cardiomyocytes infected with Ad-GFP or Ad-PRMT1 is shown by F-actinin staining (red). Scale bar: 50 μm. (F) Quantitative PCR analysis of Anp and Bnp expressions in cardiomyocytes. n = 3‒6 samples/group. Data are presented as the mean ± SE. *P < 0.05, **P < 0.01, ***P < 0.001. Furam, furamidine.

To further observe the direct effects of PRMT1 on cardiomyocytes, we treated ISO-induced H9C2 cardiomyocytes with the PRMT1-specific inhibitor furamidine. The results revealed that the protein expression levels of PRMT1 and its methylation product ADMA were significantly decreased after furamidine intervention ( Figure 2A). Compared with that of the ISO-treated H9C2 MH group, the surface area of the cardiomyocytes was further expanded after treatment with the PRMT1-specific inhibitor ( Figure 2B). In addition, the number of TUNEL-positive cardiomyocytes in the ISO-induced H9C2 group markedly increased, which further increased after furamidine intervention ( Figure 2C). Next, we treated ISO-induced H9C2 cells with PRMT1 overexpression. As shown in Figure 2D, H9C2 cells were transfected with the PRMT1-overexpressing lentivirus. Moreover, the results of the cell surface area analysis revealed that PRMT1 overexpression inhibited ISO-induced cardiomyocyte hypertrophy ( Figure 2E). Quantitative PCR analysis revealed that the mRNA expression levels of *Anp* and *Bnp* in ISO-induced H9C2 cardiomyocytes were significantly increased, and PRMT1 overexpression ameliorated these ISO-induced increases ( Figure 2F). The results described above indicated that PRMT1 inhibition and overexpression pretreatment aggravated and attenuated ISO-induced pathological changes, respectively.

### SRSF1, a downstream target of PRMT1, is involved in MH by affecting CaMKIIδ in cardiomyocytes

**Figure 3:**
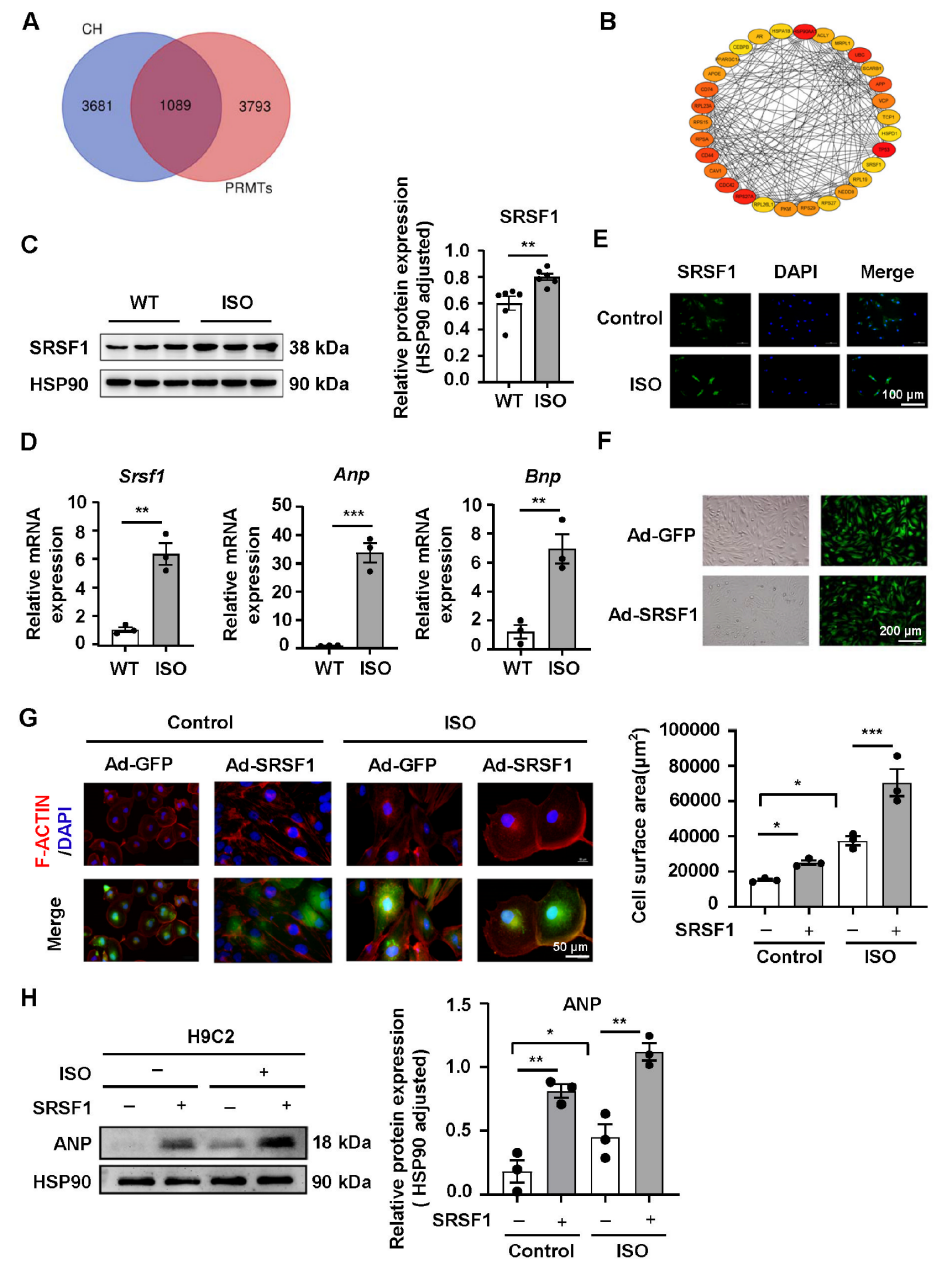
Figure 3 SRSF1, a downstream target of PRMT1, is increased in myocardial hypertrophy, and overexpression of SRSF1 exacerbates ISO-induced myocardial hypertrophy in cardiomyocytes (A) Gene intersection of PRMT1 and myocardial hypertrophy. (B) The top thirty relevant target genes are screened by cytoscape. (C) Western blot analysis of myocardial SRSF1 expression in WT and ISO-treated mice. (D) mRNA levels of myocardial Srsf1, Anp, and Bnp in WT and ISO-treated mice. (E) Representative immunofluorescence images of cardiomyocytes treated with ISO. Scale bar: 100 μm. (F) Representative immunofluorescence images of cardiomyocytes infected with Ad-GFP or Ad-SRSF1. (G) The cell surface area of cardiomyocytes infected with Ad-GFP or Ad-SRSF1 is shown by F-actinin staining (red). Scale bar: 50 μm. (H) Protein level of ANP in cardiomyocytes infected with Ad-GFP or Ad-SRSF1. n = 3‒6 samples/group. Data are presented as the mean ± SE. *P < 0.05, **P < 0.01, ***P < 0.001.

To screen for the downstream target of PRMT1 that contributes to MH, bioinformatics analysis was performed, and a potential correlation between PRMT1 and SRSF1 was observed ( Figure 3A,B). To investigate whether SRSF1 is responsible for MH, we first detected changes in SRSF1 expression in MH. As shown in Figure 3C, the protein expression level of SRSF1 in the myocardial tissues of ISO-induced MH mice was significantly higher than that in normal controls ( Figure 3C). Quantitative PCR analysis revealed that the mRNA expression levels of *Srsf1*, *Anp* and *Bnp* in ISO-induced MH mice were obviously increased ( Figure 3D). Additionally, a higher intensity of SRSF1 immunofluorescence was observed in the ISO-induced H9C2 cardiomyocyte group than in the control group ( Figure 3E). To further explore the impact of SRSF1 expression changes on MH, we transfected H9C2 cardiomyocytes with the SRSF1-overexpressing lentivirus and GFP adenovirus ( Figure 3F). Compared with that in the GFP-overexpressing group, the surface area of cardiomyocytes in the SRSF1-overexpressing group was significantly greater. Compared with that of the ISO-treated group, the cell surface area of the SRSF1-overexpressing group was further increased ( Figure 3G). Furthermore, the protein expression level of ANP in cardiomyocytes in the SRSF1-overexpressing group was markedly higher than that in the GFP-overexpressing group, and SRSF1 overexpression exacerbated the increase in ANP expression induced by ISO ( Figure 3H). Taken together, these results demonstrated for the first time that myocardial SRSF1 was elevated in MH and that SRSF1 overexpression aggravated ISO-induced cardiomyocyte hypertrophy.

**Figure 4:**
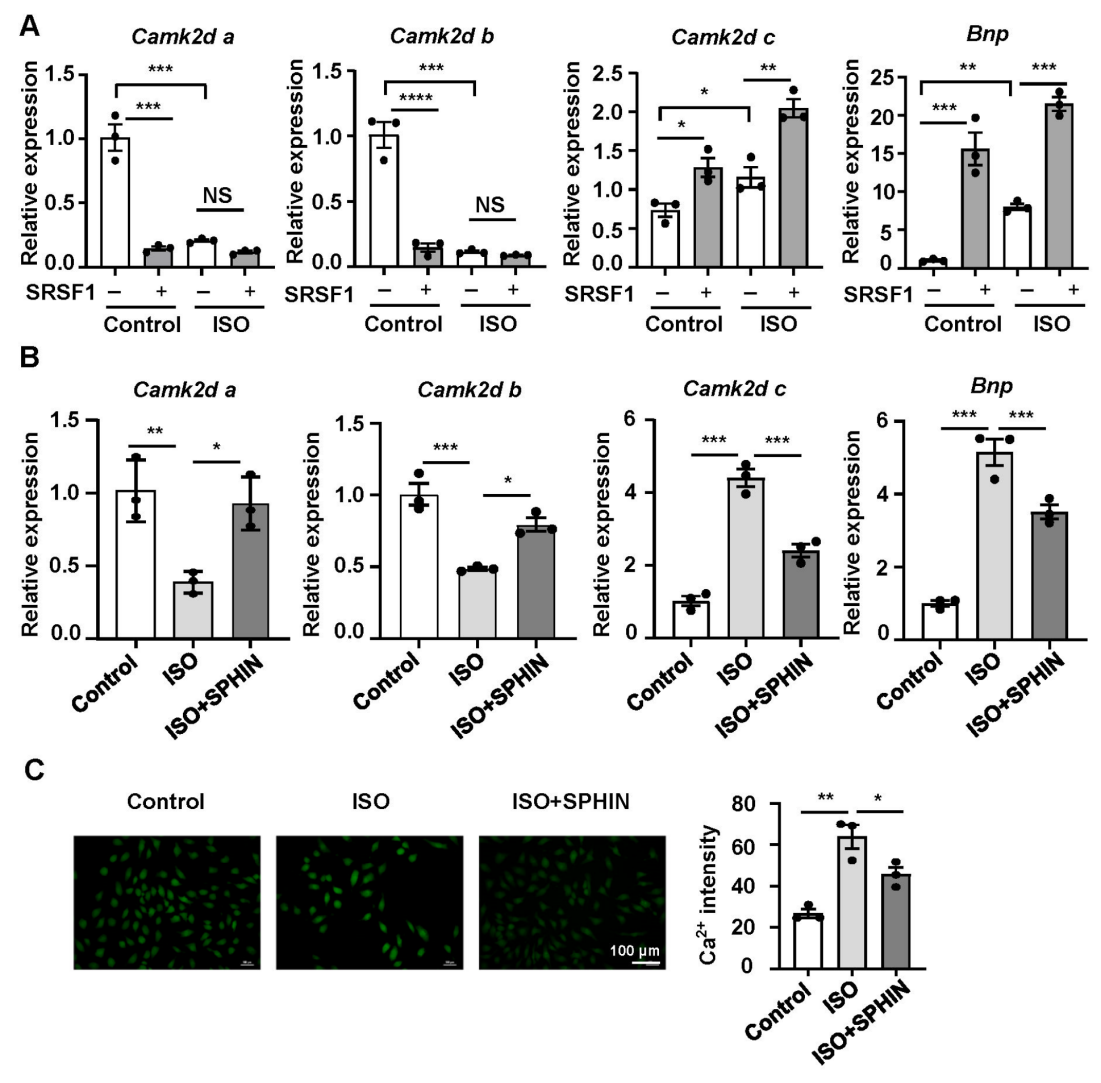
Figure 4 SRSF1 affects myocardial hypertrophy by regulating CaMKIIδ expression (A) mRNA levels of Camk2d a, Camk2d b, Camk2d c, and Bnp in cardiomyocytes transfected with Ad-GFP or Ad-SRSF1. (B) mRNA levels of Camk2d a, Camk2d b, Camk2d c, and Bnp in cardiomyocytes treated with the SRSF1 inhibitor SPHINX31. (C) Intracardiomyocyte calcium ion concentration measured by Fluo-4AM and quantification data. Scale bar: 100 μm. n = 3 samples/group. Data are presented as the mean ± SE. *P < 0.05, **P < 0.01, ***P < 0.001, ****P < 0.0001. SPHIN, SPHINX31.

Previous studies have shown that SRSF1 is specifically expressed in heart tissue and that its aberrant expression affects the alternative splicing of CaMKIIδ [10]. Therefore, in the present study, we explored whether SRSF1 induces MH through regulating CaMKIIδ. First, quantitative PCR analysis showed that the mRNA expression levels of *Camk2d a* and *Camk2d b* in ISO-induced H9C2 cardiomyocytes were significantly lower than those in control cardiomyocytes and that SRSF1 overexpression had no significant effect on *Camk2d a* and *Camk2d b* expressions in ISO-induced H9C2 cardiomyocytes. However, SRSF1 overexpression further enhanced the increase in *Camk2d c* and *Bnp* mRNA expressions caused by ISO ( Figure 4A). Additionally, the SRSF1 inhibitor SPHINX31 partially restored the decrease in *Camk2d a* and *Camk2d b* and the increase in *Camk2d c* and *Bnp* elicited by ISO ( Figure 4B). Furthermore, the results of Fluo-4AM staining demonstrated that the fluorescence intensity of calcium ions in the ISO-treated group was significantly stronger than that in the control group. Administration of the SRSF1 inhibitor SPHINX31 partially restored the high intensity of calcium ion fluorescence induced by ISO ( Figure 4C). These results strongly suggested that SRSF1 participates in MH by influencing CaMKIIδ.

### PRMT1 interacts with SRSF1 and reduces the phosphorylation level of SRSF1 via methylation

**Figure 5:**
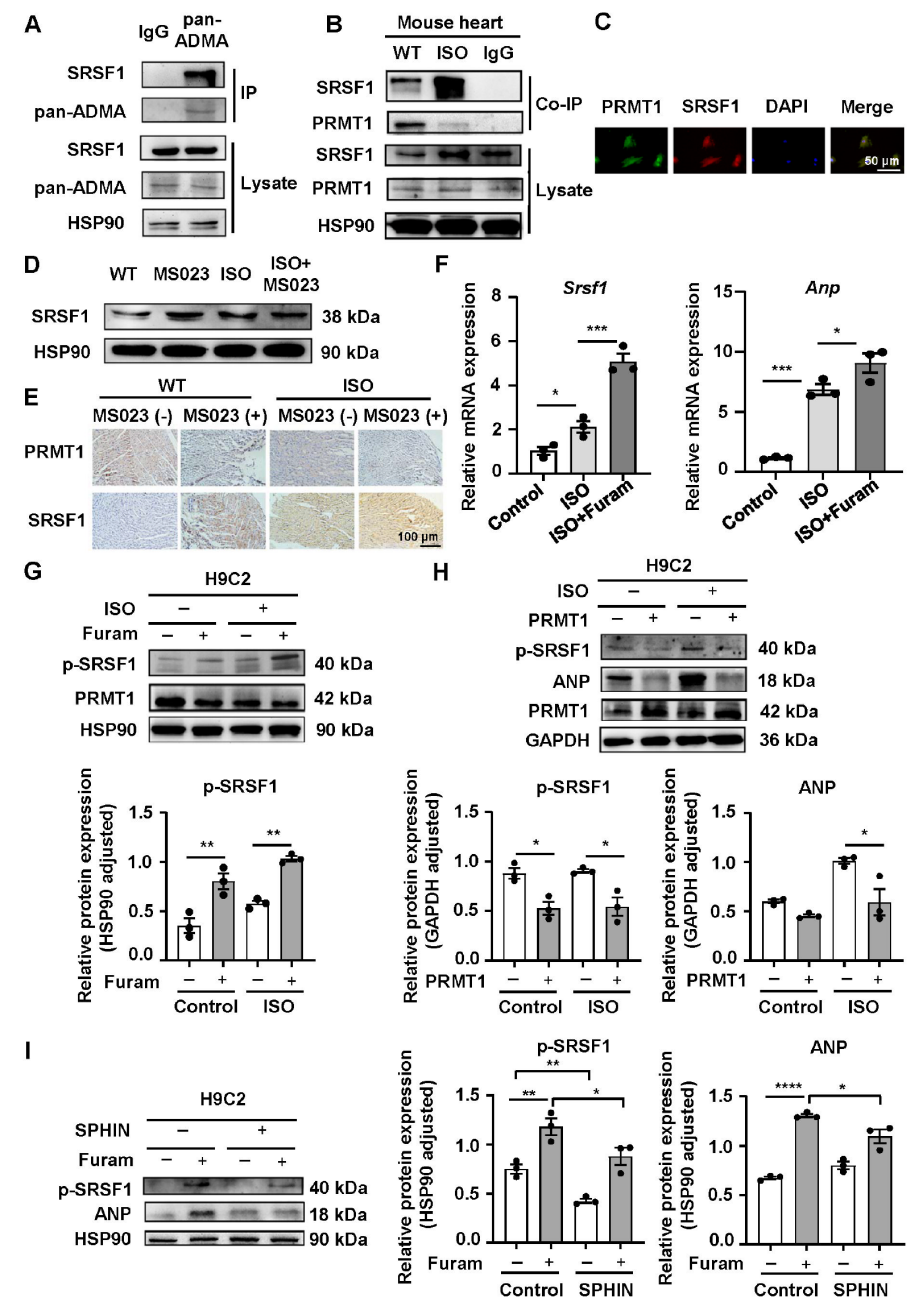
Figure 5 PRMT1 interacts with SRSF1 and reduces the phosphorylation level of SRSF1 via methylation (A) Analysis of the interaction of SRSF1 with pan-methylation in WT heart lysates. Lysates are immunoprecipitated and immunoblotted with an anti-SRSF1 antibody. (B) Analysis of the interaction of SRSF1 with PRMT1 in WT and ISO-induced heart lysates. Lysates are immunoprecipitated and immunoblotted with an anti-SRSF1 antibody. (C) Representative confocal microscopy images showing the colocalization of PRMT1 (green) and SRSF1 (red) in cardiomyocytes. Scale bar: 50 μm. (D) Western blot analysis of SRSF1 expression in WT and ISO-induced hypertrophic cardiac model mice treated with or without the PRMT1 inhibitor MS023. (E) Representative immunohistochemical images of PRMT1 and SRSF1 in WT and ISO-induced hypertrophic cardiac tissue from mice treated with or without the PRMT1 inhibitor MS023. Scale bar: 100 μm. (F) Quantitative PCR analysis of Srsf1 and Anp levels in cardiomyocytes treated with or without the PRMT1-specific inhibitor furamidine. (G) Western blot analysis of p-SRSF1 expression in cardiomyocytes treated with or without the PRMT1-specific inhibitor furamidine. (H) p-SRSF1 and ANP protein levels in cardiomyocytes infected with Ad-GFP or Ad-PRMT1. (I) Protein levels of p-SRSF1 and ANP in control or SPHINX31-treated cardiomyocytes subjected to furamidine treatment for 4 h. n = 3–6 samples/group. Data are presented as the mean ± SE. *P < 0.05, **P < 0.01, ***P < 0.001, ****P < 0.0001. Furam, furamidine; SPHIN, SPHINX31.

To confirm that PRMT1 functions via SRSF1 in MH, immunoprecipitation experiment was performed. The results revealed that SRSF1 could be methylated by PRMT1 ( Figure 5A) and that there was an interaction between PRMT1 and SRSF1 ( Figure 5B). The immunofluorescence staining results revealed the co-expression of PRMT1 and SRSF1 in the same cardiomyocytes, which further verified the interaction between SRSF1 and PRMT1 ( Figure 5C). In addition, the PRMT1 inhibitor further enhanced the increase in SRSF1 protein expression induced by ISO ( Figure 5D,E) and increased *Srsf1* and *Anp* mRNA expressions ( Figure 5F).

Next, we further explored the mechanisms by which PRMT1 and SRSF1 interact. A previous study demonstrated that the intracellular distribution of SRSF1 is affected by phosphorylation [11] and that increased SRSF1 phosphorylation promotes the translocation of SRSF1 from the cytoplasm to nuclear speckles to regulate the transcription of other genes. Because SRSF1 is rich in arginine, we postulated that PRMT1 may affect the phosphorylation of SRSF1 by methylating SRSF1. Here, after the administration of the PRMT1-specific inhibitor furamidine, the protein expression level of phosphorylated SRSF1 (p-SRSF1) was increased not only in H9C2 cardiomyocytes in the control group but also in those in the ISO-induced group ( Figure 5G). Moreover, PRMT1 overexpression not only significantly reduced the p-SRSF1 protein expression level in cardiomyocytes in the GFP group but also inhibited the protein expressions of p-SRSF1 and ANP in ISO-induced H9C2 cardiomyocytes ( Figure 5H).

We subsequently pretreated H9C2 cardiomyocytes with the SRSF1 phosphorylation inhibitor SPHINX31. As summarized in Figure 5I, the SRSF1 phosphorylation inhibitor decreased the protein expression level of p-SRSF1 and partially reversed the increase in p-SRSF1 protein expression induced by PRMT1 inhibition. The results described above indicate that PRMT1 and SRSF1 cooperate in cardiomyocytes and that PRMT1 reduces the level of SRSF1 protein phosphorylation by methylating SRSF1.

### Inhibiting the phosphorylation of SRSF1 is favourable for improving MH

**Figure 6:**
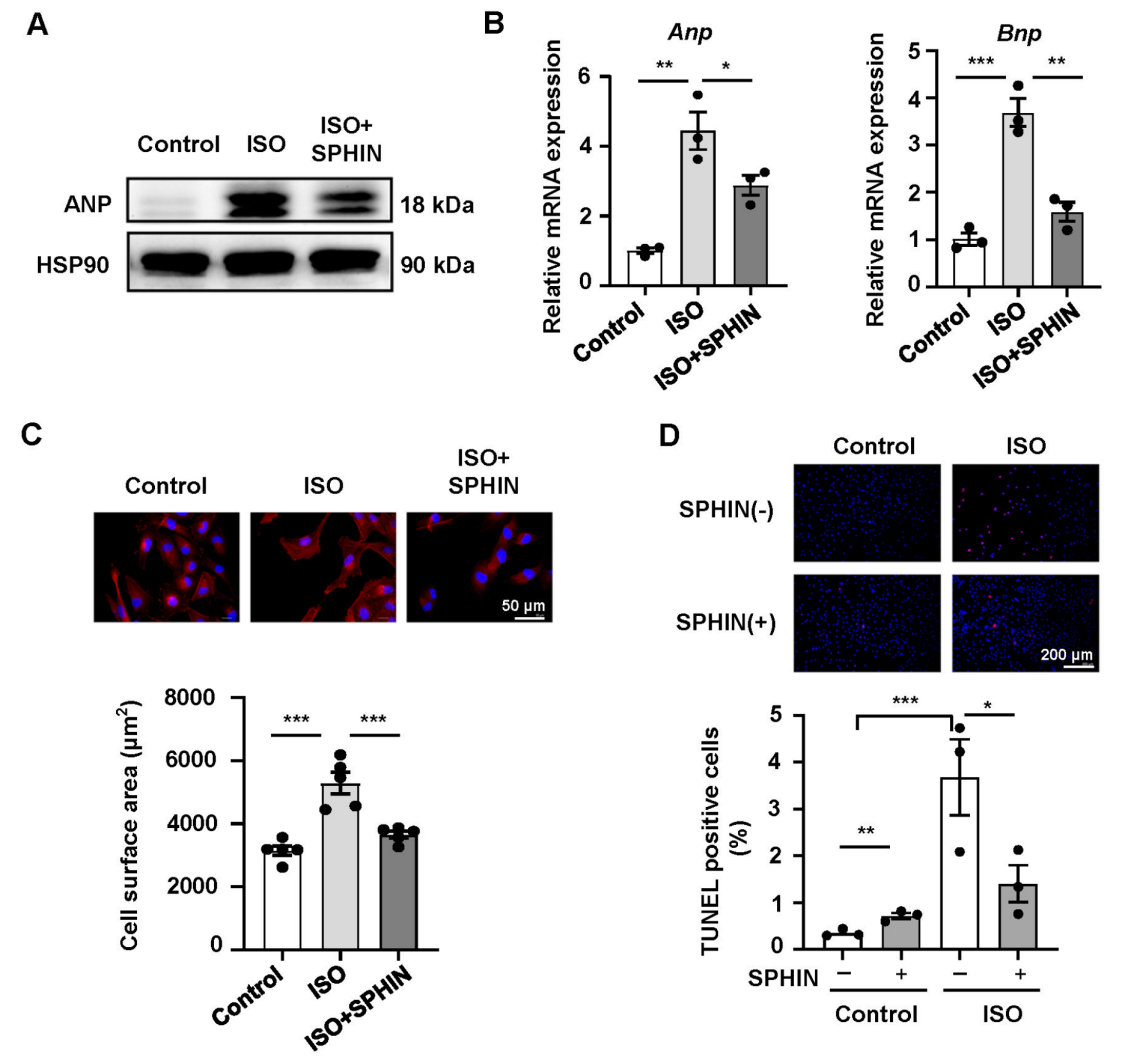
Figure 6 Inhibiting the phosphorylation of SRSF1 improves MH (A) Western blot analysis of ANP expression in cardiomyocytes treated with the SRSF1 inhibitor SPHINX31. (B) Quantitative PCR analysis of the mRNA expressions of Anp and Bnp in cardiomyocytes treated with the SRSF1 inhibitor SPHINX31. (C) Representative images of cardiomyocytes immunostained with F-actinin staining and quantification data. Scale bar: 50 μm. (D) Representative microscopy images of TUNEL-positive nuclei (red) in cardiomyocytes treated with SPHINX31 and quantification of apoptotic cells. Scale bar: 200 μm. n = 3‒5 samples/group. Data are presented as the mean ± SE. *P < 0.05, **P < 0.01, ***P < 0.001. SPHIN, SPHINX31.

Additionally, we further investigated the impact of SRSF1 phosphorylation on ISO-induced MH. The results revealed that the protein expression level of ANP in H9C2 cardiomyocytes in the SRSF1 phosphorylation inhibitor group was lower than that in the ISO-treated group ( Figure 6A). Moreover, the mRNA expression levels of *Anp* and *Bnp* in SPHINX31-treated H9C2 cells were significantly lower than those in ISO-treated H9C2 cells ( Figure 6B). In addition, SPHINX31 treatment inhibited ISO-induced expansion of the surface area of H9C2 cardiomyocytes to some extent ( Figure 6C). TUNEL staining revealed that SPHINX31 significantly reduced ISO-induced H9C2 cardiomyocyte apoptosis ( Figure 6D). These findings demonstrated that inhibiting the activity of phosphorylated SRSF1 could ameliorate ISO-induced cardiomyocyte hypertrophy.

## Discussion

We have made several novel observations in the present study. First, PRMT1 expression was reduced in myocardial tissues under MH conditions, and PRMT1 inhibition induced a hypertrophic phenotype in cardiomyocytes, while PRMT1 overexpression protected cardiomyocytes against ISO-induced pathological reactions. Second, we provided direct evidence that there is an interaction between PRMT1 and SRSF1 and that PRMT1 inhibits the phosphorylation of SRSF1 via the methylation of SRSF1. Third, we demonstrated that a low level of SRSF1 phosphorylation resulted in abnormal selective splicing of the exons in *Camk2d* mRNA, in which *Camk2d a* and *Camk2d b* mRNA expression levels were increased and *Camk2d c* mRNA expression level was decreased, thereby attenuating MH. These results strongly suggest that PRMT1 may ameliorate MH by modulating SRSF1.

MH is the consequence of the cardiac response to various traumatic stimuli and is an independent risk factor for cardiovascular diseases [12]. The process of MH development is frequently accompanied by cardiomyocyte apoptosis and myocardial fibrosis [13]. Cardiomyocyte apoptosis results in a decreased number of cardiomyocytes and reduced myocardial contractility [14]. Myocardial fibrosis decreases ventricular compliance and prevents it from effectively ejecting blood [15]. Progressive aggravation of reduced myocardial contractility and myocardial fibrosis eventually leads to HF, malignant arrhythmia, or even sudden death [16]. In the present study, we successfully established an MH model via ISO. As a β-adrenoceptor agonist, ISO enhances myocardial contractility by binding to receptors and further induces MH [ 17, 18]. In ISO-treated mice, ANP protein expression in myocardial tissues was increased, LV contractility was weakened, EF and FS were reduced significantly, the HW/BW ratio was increased significantly, and LV cardiomyocytes were enlarged morphologically. Moreover, these pathophysiological changes were accompanied by increased myocardial fibrosis and cardiomyocyte apoptosis.

Epigenetic modification is an important factor contributing to MH [19]. PRMTs regulate arginine methylation in proteins through three different mechanisms: monomethyl arginine (MMA), asymmetric dimethylarginine (ADMA) and symmetrical dimethylarginine (SDMA) [20]. PRMT1, the earliest discovered PRMT and the predominant type I PRMT in mammalian cells, accounts for more than 85% of cellular PRMT activity [21]. In our study, we found that myocardial PRMT1 expression was reduced in MH models and that PRMT1 inhibition aggravated MH, whereas PRMT1 overexpression had the opposite effect. These findings suggest that PRMT1 plays a crucial regulatory role in MH, which is consistent with previous findings that knocking out cardiac-specific *PRMT1* could induce the MH phenotype accompanied by a series of symptoms, including deterioration of myocardial fibrosis and impaired cardiac function [6].

PRMT1 methylates a variety of histone and nonhistone proteins to regulate multiple cellular functions. Nonhistone proteins methylated by PRMT1 are divided into four categories according to the molecular function of the substrate, namely, transcription factors, RNA-binding proteins, DNA damage repair factors, and proteins that play roles in signal transduction and other functions [22]. SRSF1, a prototypical splicing factor, is a type of RNA-binding protein [23]. In the present study, we used Gene Expression Omnibus (GEO), various bioinformatics methods, and IP experiments to verify that SRSF1 is a downstream protein of PRMT1 and participates in the biological process by which PRMT1 improves MH. SRSF1, the first identified member of the serine/arginine-rich family [24], contains an RNA recognition motif at the N-terminus that controls the selective binding of RNA and a serine/arginine-rich domain at the C-terminus that modulates protein interactions [25]. Substantial evidence has shown that SRSF1 is involved in cancer, neurodegenerative disease, and autoimmune disease through the targeted regulation of the selective splicing of multiple genes [26]. SRSF1 mainly plays multiple roles in mRNA transcription, splicing, nuclear export, decay, and translation [25]. SRSF1 plays a critical role in the alternative splicing of *Camk2d* [27].

CaMKIIδ is mostly associated with processes such as myocardial remodeling [28], arrhythmias [29], interstitial fibrosis [30], and apoptosis [31]. Studies have shown that all *Camk2d* genes can be affected by alternative splicing. *Camk2d* mRNA contains multiple exons. Different splicing forms translate different CaMKIIδ subtypes via different transcripts [32]. CaMKIIδ A, CaMKIIδ B and CaMKIIδ C are located in the heart [33]. In our study, SRSF1 reduced the mRNA expression levels of *Camk2d a* and *Camk2d b* and increased the mRNA expression level of *Camk2d c* in the control group. In the ISO group, SRSF1 increased the mRNA expression level of *Camk2d c*, which is consistent with the finding that high CaMKIIδ C expression promotes MH [34]. However, SRSF1 did not significantly affect the mRNA expression of *Camk2d a* or *Camk2d b* in the ISO group. This may be related to the fact that ISO has already resulted in significant decreases in the mRNA expression of *Camk2d a* and *Camk2d b* or may be associated with ISO-induced alterations in SRSF1 splicing.

The splicing function of SRSF1 is closely correlated with SRSF1-dependent exon skipping through the splicing of exons and introns at both ends. By specifically binding with exonic splicing enhancers (ESEs), SRSF1 promotes U2AF to identify the 3′ splicing site and U1 to bind the exon downstream of the 5′ splicing site [10]. The selective splicing of exons 14, 15 and 16 in *Camk2d* mRNA by SRSF1 results in three mRNA subtypes: *Camk2d a*, *Camk2d b*, and *Camk2d c*, which can translate into three protein subtypes: CaMKIIδ A, CaMKIIδ B, and CaMKIIδ C [27]. These CaMKIIδ subtypes play dissimilar roles in cardiomyocytes. CaMKIIδ A induces MH by enhancing the L-type calcium current of neonatal cardiomyocytes, but this phenomenon does not occur in adult cardiomyocytes [35]. CaMKIIδ B improves MH by increasing the level of the mitochondrial Ca ^2+^ uniporter (MCU). CaMKIIδ C elicits cardiomyocyte apoptosis and mitochondrial dysfunction [36].

Our further research demonstrated that treatment with an SRSF1 phosphorylation inhibitor increased the mRNA expression levels of *Camk2d a* and *Camk2d b* and decreased the mRNA expression level of *Camk2d c* in ISO-induced H9C2 cardiomyocytes, suggesting that selective *Camk2d* splicing is inseparable from the phosphorylation of SRSF1. In addition, SRSF1 exerts its function mainly in the nuclear speckle, which depends on the degree of protein phosphorylation [37]. Serine-arginine protein kinase 1 (SRPK1) binds with SRSF1 at high affinity and phosphorylates serine and arginine in the N-terminus of its serine/arginine-rich domain, thus facilitating nuclear import and localization in the nuclear speckle [38].

Additionally, we found that PRMT1 could modulate the phosphorylation level of SRSF1. The serine/arginine-rich domain in SRSF1 can be phosphorylated and methylated [25], and high methylation levels affect the phosphorylation level of SRSF1 [39]. Studies have demonstrated that nonhistone protein methylation affects its phosphorylation. For example, arginine methylation of the transcription factor Forkhead box O (FOXO) inhibits the phosphorylation of FOXO by Akt [40]. However, the underlying mechanism by which protein methylation affects protein phosphorylation remains unclear, probably because the adjacent phosphorylation site is disrupted owing to changes in the protein spatial structure after methylation or because its binding to phosphate kinases is affected.

**Figure 7:**
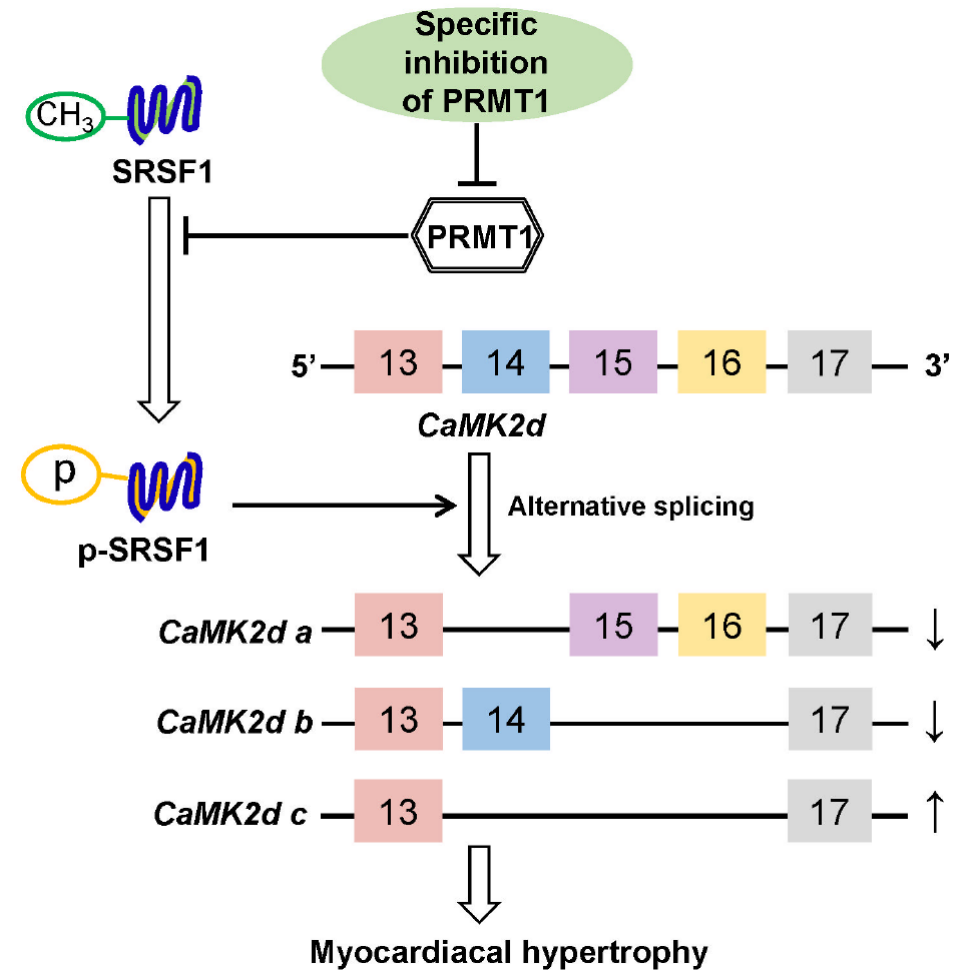
Figure 7 Schematic diagram of the mechanism by which PRMT1 alleviates myocardial hypertrophy PRMT1 alleviates cardiac hypertrophy by increasing the expressions of CaMK2d a and CaMk2d b and decreasing the expression of CaMK2d c through reducing the phosphorylation level of SRSF1 via methylation.

In summary, we explored the potential interactions among PRMT1, SRSF1, and CaMKIIδ in MH and found that the methylation of SRSF1 by PRMT1 reduces its phosphorylation level, which further changes its selective splicing function on *Camk2d*, resulting in increased *Camk2d a* and *Camk2d b* mRNA expressions and decreased *Camk2d c* mRNA expression, and eventually alleviating MH ( Figure 7). Nevertheless, these findings not only are scientifically important but also form the foundation of the search for an alternative therapy to prevent MH.
